# NT-proBNP independently predicts long term mortality after acute exacerbation of COPD – a prospective cohort study

**DOI:** 10.1186/1465-9921-13-97

**Published:** 2012-10-29

**Authors:** Arne Didrik Høiseth, Torbjørn Omland, Tor-Arne Hagve, Pål H Brekke, Vidar Søyseth

**Affiliations:** 1 Division of Medicine, Akershus University Hospital and Institute of Clinical Medicine, University of Oslo, Oslo, Norway; 2 Unit of Medical Biochemistry, Division of Diagnostics and Technology, Akershus University Hospital and University of Oslo, Oslo, Norway; 3 Akershus University Hospital, 1478, Lørenskog, Norway

**Keywords:** Chronic obstructive pulmonary disease, Heart failure, NT-proBNP, Mortality

## Abstract

**Background:**

Cardiovascular disease is prevalent and frequently unrecognized in patients with chronic obstructive pulmonary disease (COPD). NT-proBNP is an established risk factor in patients with heart failure. NT-proBNP may also be released from the right ventricle. Thus serum NT-proBNP may be elevated during acute exacerbations of COPD (AECOPD). The prognostic value of NT-proBNP in patients hospitalized with AECOPD is sparsely studied. Our objective was to test the hypothesis that NT-proBNP independently predicts long term mortality following AECOPD.

**Methods:**

A prospective cohort study of 99 patients with 217 admissions with AECOPD. Clinical, electrocardiographic, radiological and biochemical data were collected at index and repeat admissions and analyzed in an extended survival analysis with time-dependent covariables.

**Results:**

Median follow-up time was 1.9 years, and 57 patients died during follow-up. NT-proBNP tertile limits were 264.4 and 909 pg/mL, and NT-proBNP in tertiles 1 through 3 was associated with mortality rates of 8.6, 35 and 62 per 100 patient-years, respectively (age-adjusted log-rank p<0.0001). After adjustment for age, gender, peripheral edema, cephalization and cTnT in a multivariable survival model, the corresponding hazard ratios for dying were 2.4 (0.95-6.0) and 3.2 (1.3-8.1) (with 95% confidence intervals in parentheses, p-value for trend 0.013).

**Conclusions:**

NT-proBNP is a strong and independent determinant of mortality after AECOPD.

## Background

Comorbidities are important determinants of outcome and quality of life of patients with chronic obstructive pulmonary disease (COPD) [1]. The extrapulmonary manifestations of COPD include cardiovascular diseases such as cerebral stroke, myocardial infarction (MI), heart failure, and cardiac arrhythmias. These conditions are more common among COPD patients than in the general population, also after adjusting for smoking and other important confounders [2-6]. Moreover, heart disease often remains undiagnosed in these patients [7-10]. This may in part be because the symptoms and signs of MI and heart failure may mimic an acute exacerbation of COPD (AECOPD), but also because the classic symptom and sign of MI, i.e. chest pain and ECG changes, are poorly associated with myocardial injury during AECOPD [11-13]. Using a highly sensitive assay, we have previously shown that nearly three quarters of patients admitted with AECOPD have cardiac troponin T (cTnT) above the 99^th^ percentile (14 ng/L), and that even modest elevation is associated with increased mortality [14,15].

The cardiac peptides B-type natriuretic peptide (BNP) and the N-terminal fragment of its prohormone pro-BNP, i.e. NT-proBNP, are both established biomarkers of heart failure, [16] and are primarily used for diagnosis, risk stratification and management of heart failure. The natriuretic peptides (NP) have, however, also been shown to be elevated in patients with COPD without known heart failure [9,15,17]. It has been proposed that the NP may originate from both left and right heart in this setting [17,18]. Cor pulmonale, secondary pulmonary hypertension, or hypoxemia may represent important stimuli for the release of NP from the right heart. In stable COPD patients, increased concentration of a NP seems to be associated with poorer long-term survival, [19-22] although this finding not always is reproduced [23]. In one study of 140 stable COPD patients recruited at an outpatient clinic, NT-proBNP levels were associated with mortality in unadjusted analysis, but not after adjustment for echocardiographic variables, suggesting that underlying heart disease may be the cause of both increased NT-proBNP and mortality [21].

During AECOPD the NP levels are higher than in the stable state, [18,23,24] and the stimuli for their release may be more complex. Three papers addressing NP as prognostic markers after AECOPD do not conclude uniformly, [15,17,18] as only Medina found significant association with long-term mortality. Consequently, it remains unresolved whether circulating natriuretic peptides measured during AECOPD provide independent long-term prognostic information. Moreover, the potential incremental prognostic value of combining cardiac troponins, using contemporary high sensitivity assays, and NP still remains unresolved. The release of the two biomarkers may in part be due to the same mechanisms, but their levels during exacerbations have shown conflicting degrees of correlation [15,25]. In a single study only have both NT-proBNP and cTnT during AECOPD been measured and related to survival [15]. cTnT, measured by a former generation assay, and NT-proBNP above the upper limits of normal were both associated with increased 30 days mortality, but not with 12 month mortality. Further, while NT-proBNP elevation provided incremental prognostic information to cTnT, the converse was not true.

We hypothesized that the concentrations of NT-proBNP and cTnT on admission for exacerbation of AECOPD, as measured by a highly sensitive assay (hs-cTnT), provide independent prognostic information about long-term mortality.

## Methods

### Material

During 23 months in 2005 and 2006, all patients admitted with AECOPD were eligible for inclusion in this prospective cohort study. If patients had more than one admission during the inclusion period, data from all admissions were recorded and used in the analyses, as described in the statistics section. On each admission we recorded clinical data, ECGs, chest radiographs, and results from blood analyses. The clinical data recorded were heart rate, blood pressure, body temperature, respiratory rate, arterial blood gases (pH, PaCO_2,_ PaO_2_), arterial oxygen saturation (SaO_2_), wheezing, chest pain, and the use of accessory muscles of respiration. ECGs were analyzed for the presence of P pulmonale, QRS axis deviation, signs of acute ischemia (ST-segment depression or elevation), and previous MI (T-wave inversion, pathological Q-wave, loss of R, or left bundle branch block, and by using the cardiac infarction injury score (CIIS), where a score ≥20 indicates high probability of prior MI) [26]. Two independent investigators, who were blinded to all clinical data, conducted the ECG analyses. Differences were settled by consensus regarding the qualitative analyses, and by a third investigator regarding CIIS (when the two initial scorings were on opposite sides of 20). Two blinded investigators examined the radiographs for the presence of cephalization of the lung veins, infiltrates, cardiomegaly, or pleural effusions.

Haemoglobin, leucocyte, neutrophil and platelet counts, electrolytes, blood glucose, and serum CRP were recorded from the hospital records. Serum and plasma from blood drawn on admission were stored at −80°C for subsequent analysis of creatinine, hs-cTnT and NT-proBNP (Roche Diagnostics, Mannheim, Germany). The NT-proNBP assay has a lower limit of detection of 5.0 pg/mL. The coefficients of variation are reported to be 4.2%, 2.4% and 1.3% at concentrations of 44 pg/mL, 126 pg/mL and 2410 pg/mL, respectively. Body mass index (BMI) and spirometry during stable phase were recorded when available. Medical history was obtained by patient interview and by retrieving information from hospital records. Patients were categorized as current, former or never smokers. We also recorded the number of admissions with AECOPD during the 12 months prior to inclusion. The endpoint was length of survival after the initial admission, with end of follow-up on December 31^st^ 2008. Survival status and dates of death were retrieved from the National Population Registry.

All included patients gave written informed consent. The study protocol was approved by the Data Inspectorate and by the Regional Committee for Research Ethics. Additional information regarding the inclusion procedure and the study population is available in the additional material [see Additional file 1 and an earlier paper [14].

### Statistical analyses

First, NT-proBNP was categorized by tertiles, and the mortality rates between the NT-proBNP tertiles were compared using age-adjusted log-rank test. Investigation of potential confounding was done in two steps: First by identifying variables associated with NT-proBNP, and then by analyzing which of these that were also associated with mortality. Univariable baseline associations between NT-proBNP tertiles and covariables were analyzed using Chi-square or Fisher’s exact test for categorical variables and Kruskall-Wallis test for continuous variables. Then, for the covariables that were associated with NT-proBNP with a p-value<0.20, we investigated the association between the corresponding covariable and mortality using an age-adjusted log-rank test. In this analysis, continuous variables were categorized: Number of admissions during the past year was categorized as 0, 1 or ≥2, as in the ECLIPSE study [27]. hs-cTnT was categorized into three groups according to cut-offs at 14 and 40 ng/L, as these cut-offs have shown to discriminate well with regard to survival [14]. The remaining continuous covariables were dichotomized at pre-specified limits: FEV_1_/FVC at the mean (0.45), BMI at the lower limit of normal (20 kg/m^2^), CRP at 50 mg/L, and creatinine at 100 μmol/L.

The variables associated with both NT-proBNP and mortality (p<0.20 in both tests above) were included in a multivariable survival analysis. For these variables, we also investigated potential effect modification and tested for homogeneity using Mantel-Haenszel test [28]. If this indicated effect modification of the association between NT-proBNP and mortality, a product-term between NT-proBNP and the corresponding covariable was included in the initial multivariable survival model. If the product-term proved significant with p-value<0.05, the covariable in question was considered an effect modifier.

Up until this point, only the index observations were used, but for the final survival analysis, we used an extended Cox regression analysis with time-dependent covariables, [29] such that covariables that may change between admissions (i.e. all covariables except age at inclusion, gender, lung function, BMI, and medical history) were updated at each subsequent admission. Using a backward elimination procedure, the model was reduced by removing variables with p-values >0.05 provided that the coefficient of the association between mortality and NT-proBNP changed less than 20%. Age and gender were kept in the model by convention. Finally, the interaction terms between NT-proBNP and age, gender, and creatinine were added to the model to check whether important interactions had been overlooked.

We performed four supplementary survival analyses: i) The described analysis restricted to the patients with no history of heart failure, ii) with NT-proBNP as a continuous variable (log-transformed; lnBNP), iii) with NT-proBNP categorized at 1000 and 2500 pg/mL, as suggested by Abroug et al., [30] and iv) the described analysis restricted to patients without chest pain or ECG changes indicative of acute ischemia (i.e. patients that may fulfill the criteria for an MI).

The proportional hazards assumption was checked by the Martingale residuals on standard Cox regression models using the index observations only. Analyses of effect modification were done using STATA 10 (Stata Corp LP, TX, USA). All other analyses were done using SAS 9.2 (SAS Institute Inc., Cary, NC, USA).

## Results

99 patients were included. Mean age at inclusion was 71.5 years (standard deviation (SD) 9.0 years), and 47 (47%) were female. Spirometry measurement from the stable state was available in 88 patients. Mean FEV_1_ was 0.91 liter (SD 0.45 liter), and mean FEV_1_/FVC was 45.3% (SD 14.2%). Median NT-proBNP concentration at inclusion was 423.3 pg/mL, and the tertile limits were 264.4 and 909 pg/mL. 41 patients were readmitted during the inclusion period, adding to a total of 219 admissions. In 217 of these, we had NT-proBNP measured, with a median of 560.4 pg/mL. Among the patients with more than one NT-proBNP measurement, the median change (absolute value) from the previous measurement was 66% (IQR 41-150%). Three patients had two consecutive NT-proBNP measurements less than 5% apart, but none of these changed tertiles. The prevalence of categorical variables and the mean of continuous variables in each tertile of NT-proBNP at inclusion are reported in Table 1 for those variables associated with NT-proBNP (p<0.20). Among the variables not associated with NT-proBNP were: a history of coronary artery disease (27%), arterial hypertension (31%), smoking status (48% current smokers), chest pain on admission (10%), kidney function (mean creatinine 76 μg/L), P pulmonale (25%), right axis deviation (10%), atrial fibrillation (7%), prior MI (40%), and ischemia in ECG (27%). Median NT-proBNP concentrations (with interquartile range) among patients with 0, 1 and ≥2 admissions the past 12 months were 339 (183–1054), 679 (154–1438) and 421 (157–1389) pg/mL, respectively (p=0.826). The correlation between NT-proBNP and hs-cTnT was moderate (both logarithmically transformed, Pearson rho=0.34, p=0.0006).

**Table 1:** Prevalence or mean of relevant covariables in each tertile of NT-proBNP at baseline

	**NT-proBNP, pg/mL**			**p-value**
	**Tertile 1 (< 264.4)**	**Tertile 2 (264.4-909)**	**Tertile 3 (≥909)**	
Female, n (%)	16 (48)	18 (55)	13 (39)	0.463
Age (years) , mean (SD)	66.0 (7.4)	71.9 (8.2)	76.6 (8.1)	<0.0001
BMI (kg/m^2^), mean (SD)	23.2 (5.3)	23.6 (6.1)	21.3 (3.9)	0.149
Medical history				
FEV_1_/FVC, mean (SD)^*^	0.46 (0.16)	0.45 (0.14)	0.46 (0.13)	0.943
FEV_1_ (L), mean (SD)^*^	0.99 (0.55)	0.81 (0.36)	0.94 (0.39)	0.305
FEV_1_ (% of pred.), mean (SD)*	33 (18)	31 (20)	36 (20)	0.375
Heart failure, n (%)	3 (9.1)	4 (12)	7 (21)	0.445
Diabetes Mellitus, n (%)	3 (9.1)	5 (15)	0	0.008
Atrial fibrillation, n (%)	1 (3.0)	3 (9.1)	6 (18)	0.149
Admissions past 12 months				0.814
0	13 (35)	14 (38)	10 (27)	
1	8 (31)	7 (27)	11 (42)	
≥2	12 (33)	12 (33)	12 (33)	
Findings on admission				
Peripheral edema, n (%)	3 (9.1)	6 (18)	9 (27)	0.179
Cephalization, n (%)	2 (6.1)	3 (9.1)	11 (33)	0.007
SaO_2_ (%), mean (SD)	92.1 (3.0)	89.0 (6.8)	88.1 (9.0)	0.177
Biochemistry				
CRP (mg/L), median (IQR)	20 (2.0-35)	38 (17–71)	54 (2.0-84)	0.071
hs-cTnT (ng/L), median (IQR)	14 (8.3-26)	31 (15–41)	44 (22–58)	0.0006
Creatinine (μmol/L), median (IQR)	64 (55–79)	65 (53–89)	75 (60–91)	0.213

The table includes variables associated with NT-proBNP tertiles (p<0.20) with past admissions, lung and kidney function added to better characterize the cohort. * Spirometry available in 88 patients (n=28, 28 and 29 in tertiles 1–3). SD, standard deviation; IQR, interquartile range; BMI, body mass index; CRP, C-reactive protein; hs-cTnT, high-sensitivity cardiac troponin T.

N=99 unless otherwise specified.

**Figure 1:**
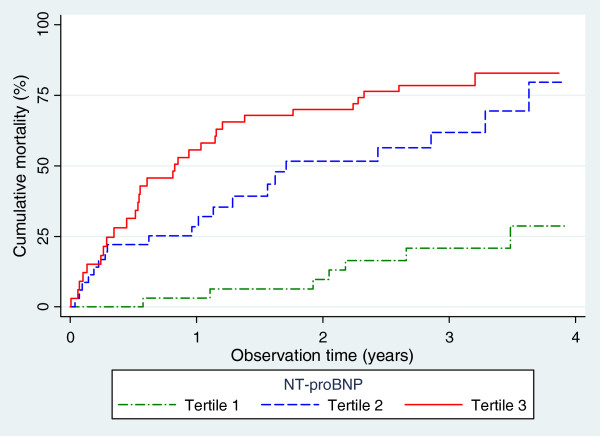
**Mortality after acute exacerbation of COPD stratified by level of NT-proBNP.** Based on 217 admissions and 57 mortalities in 99 patients.

During a median follow-up of 1.9 years, 57 (58%) patients died, with an overall mortality rate of 28.7 per 100 patient-years. NT-proBNP tertiles on inclusion were associated with increasing mortality rates, i.e. 8.6 (95% confidence interval 4.3-17), 35 (23–54) and 62 (43–91) per 100 patient-years, respectively (Figure 1, age-adjusted log-rank p-value<0.0001). When applying the cut-offs suggested by Abroug, [30] the mortality rates were 19, 25 and 120 per 100 patient-years among patients with NT-proBNP <1000, 1000–2500 and ≥2500 pg/mL, respectively (age-adjusted log-rank p-value<0.0001).

Of the variables that were associated with NT-proBNP concentrations, history of diabetes and lung function failed to show any association with mortality and were therefore not included in further analyses. For the remaining covariables, stratified analyses of mortality through the tertiles of NT-proBNP were then performed (Table 2). Mantel-Haenszel tests showed no statistically significant effect modification of the association between NT-proBNP and mortality by any of the covariables listed in Table 2, and the following variables were included in the initial multivariable model: age, gender, BMI, history of heart failure, atrial fibrillation on admission, peripheral edema, cephalization of the lung veins, CRP, and hs-cTnT. Reduction of the model gave the final model in Table 3, showing increasing mortality with increasing levels of NT-proBNP (p =0.013 for trend). hs-cTnT, gender, peripheral edema, and cephalization of the lung veins were also independently associated with mortality. None of the interaction terms investigated proved statistically significant (p-values >0.05). Using the natural logarithm of the NT-proBNP concentration (lnBNP) as a continuous variable in the final model showed a hazard ratio of 1.5 (95% CI: 1.2-1.9, p=0.0005) per unit increase in lnBNP. By adhering to the model reduction described, BMI and CRP were eliminated from the model both when analyzed as continuous and dichotomized variables. Similarly, analyzing hs-cTnT continuously and categorized made no noteworthy change in the model. Patients with a history of heart failure had non-significantly higher NT-proBNP concentrations (median 1554 vs. 400 pg/mL, p=0.102), and when restricting the analysis to the 85 patients without history of heart failure, the associations with mortality remained practically unchanged (Table 3). At the index admission, patients with (n=35) and without (n=64) chest pain or ECG signs of acute ischemia had median NT-proBNP concentrations 655 (IQR 197–1311) and 345 (IQR 145–1312) pg/mL, respectively (p-value 0.219). When restricting the analysis to admissions without chest pain or ST-T changes, the association between cTnT and mortality increased markedly while the association between NT-proBNP and mortality was moderately attenuated and was no longer statically significant (Table 3).

**Table 2:** Mortality (m), mortality rate (MR, per 100 patient-years) in NT-proBNP tertiles, and mortality rate ratio per tertile increase in NT-proNBP

	**NT-proBNP (pg/mL)**			**Mortality Rate Ratio**	
	**Tertile 1 (< 264.4)**	**Tertile 2 (264.4-909)**	**Tertile 3 (≥909)**	**Unadjusted**	Adjusted*
	**m (MR)**	**m (MR)**	**m (MR)**		
Gender					2.4 (1.8-3.3)
Female	6 (16.5)	12 (35)	12 (67)	2.1 (1.3-3.3)	
Male	1 (1.9)	8 (39)	18 (51)	2.8 (1.8-4.2)	
Age, years					2.3 (1.6-3.2)
<65	1 (5.4)	1 (70)	1 (15)	1.8 (0.49-6.8)	
65-74	5 (7.9)	11 (33)	10 (65)	3.3 (1.9-5.6)	
≥75	1 (11)	8 (39)	19 (60)	1.7 (1.0-2.8)	
BMI, kg/m^2^					2.3 (1.7-3.2)
<20	2 (9.6)	8 (45)	11 (64)	2.1 (1.2-3.5)	
≥20	5 (7.2)	9 (30)	18 (50)	2.5 (1.6-3.7)	
History of DM					2.4 (1.7-3.2)
No	6 (7.6)	16 (33)	29 (57)	2.4 (1.7-3.3)	
Yes	1 (8.8)	4 (21)	1 (44)	2.6 (0.8-8.4)	
Cephalization					2.0 (1.4-2.7)
No	7 (8.0)	19 (37)	13 (31)	1.9 (1.3-2.8)	
Yes	0	1 (30)	17 (150)	2.2 (1.2-4.2)	
Atrial fibrillation					2.3 (1.7-3.1)
No	7 (8.0)	19 (36)	24 (50)	2.3 (1.6-3.2)	
Yes	0	1 (41)	6 (109)	2.3 (0.95-5.6)	
Peripheral edema					2.3 (1.7-3.1)
No	5 (6.4)	15 (32)	17 (38)	2.2 (1.5-3.2)	
Yes	2 (17)	5 (68)	13 (146)	2.4 (1.5-4.1)	
SaO_2_ <90%					2.3 (1.7-3.1)
No	5 (6.8)	12 (33)	17 (50)	2.5 (1.7-3.8)	
Yes	2 (12)	8 (42)	13 (68)	1.9 (1.2-3.2)	
CRP, mg/L					2.4 (1.7-3.2)
<50	6 (8.2)	13 (50)	13 (38)	2.0 (1.4-3.1)	
≥50	1 (6.0)	7 (24)	17 (90)	3.0 (1.8-5.1)	
hs-cTnT, ng/L					1.8 (1.3-2.5)
<14	2 (4.3)	1 (10)	0	0.83 (0.18-3.9)	
14-40	4 (12)	10 (30)	10 (71)	2.5 (1.5-4.4)	
≥40	1 (10)	9 (78)	20 (67)	1.5 (0.94-2.3)	
All, crude MRR	7 (7.8)	20 (36)	30 (56)	2.4 (1.7-3.2)	

Based on 217 admissions and 57 mortalities among 99 patients. Mortality rate ratio expressed as score test for trend for a one tertile increase in NT-proNBP *Adjusted for the corresponding covariable. BMI, body mass index; DM, diabetes mellitus; CRP, C-reactive protein; hs-cTnT, high-sensitivity cardiac troponin T.

**Table 3:** Hazard ratios for dying after admission for acute exacerbation of COPD

***Variable***	All patients*				***Patients with no history of heart failure†***		***Patients with no chest pain or ischemia‡***	
	***Univariate***		***Adjusted***		***Adjusted***		***Adjusted***	
	***Hazard ratio (95% CI)***	***p-value***	***Hazard ratio (95% CI)***	***p-value***	***Hazard ratio (95% CI)***	***p-value***	***Hazard ratio (95% CI)***	***p-value***
Age, per 5 years	1.3 (1.08-1.5)	0.003	0.99 (0.83-1.2)	0.872	0.95 (0.78-1.2)	0.643	0.98 (0.75-1.3)	0.860
Female	1.4 (0.81-2.3)	0.252	2.0 (1.1-3.6)	0.020	1.7 (0.87-3.3)	0.119	2.8 (1.2-6.8)	0.020
Peripheral edema	3.2 (1.9-5.5)	<0.0001	2.1 (1.2-3.8)	0.014	2.1 (1.0-4.2)	0.044	2.6 (1.1-6.1)	0.032
Cephalization	4.5 (2.5-8.0)	<0.0001	2.2 (1.2-4.2)	0.014	2.2 (1.0-4.8)	0.049	3.4 (1.2-9.8)	0.022
hs-cTnT, ng/L		p for trend 0.0001		p for trend 0.004		p for trend 0.008		p for trend 0.004
<14	1		1		1		1	
14-40	6.3 (1.9-21)	0.003	4.3 (1.2-15)	0.024	4.5 (1.3-16)	0.021	9.4 (1.2-77)	0.036
≥40	12 (3.5-38)	<0.0001	6.5 (1.8-24)	0.005	6.4 (1.7-24)	0.007	16 (1.7-140)	0.013
NT-proBNP, tertile		p for trend <0.0001		p for trend 0.013		p for trend 0.030		p for trend 0.211
1(< 264.4 pg/mL)	1		1		1		1	
2 (264.4-909 pg/mL)	4.3 (1.8-10)	0.0009	2.4 (0.95-6.0)	0.064	2.7 (1.0-7.4)	0.049	2.1 (0.60-7.5)	0.247
3 (≥ 909 pg/mL)	6.9 (3.0-16)	<0.0001	3.2 (1.3-8.1)	0.012	3.3 (1.2-9.1)	0.023	2.5 (0.59-9.0)	0.167

*Based on 217 admissions and 57 mortalities among 99 patients in an extended Cox analysis with time dependent covariables. †Based on 185 admissions among 85 patients (46 mortalities). ‡Based on 137 admissions among 64 patients (30 mortalities). hs-cTnT, high-sensitivity cardiac troponin T.

None of the models violated the proportional hazards assumption.

## Discussion

The new and important findings of the current study are that higher levels of NT-proBNP during AECOPD are associated with increased long-term mortality, and that this association is independent of important clinical, radiographic, pulmonary function, and biochemical, variables, including cTnT. Although we observed a moderately strong association between NT-proBNP and cTnT, NT-proBNP provided prognostic information above and beyond cTnT.

The prevailing view is that the primary stimulus for NT-proBNP release is myocardial strain in the face of increased right or left ventricular filling pressures [31,32]. Additionally, it has been shown that hypoxia stimulates the natriuretic peptide system [33]. Of these, both hypoxia and increased pressure in the right heart may be present in COPD, both in stable state and during exacerbations. Although there was a trend against higher concentrations of NT-proBNP with lower SaO_2_, this association was not statistically significant. In the absence of echocardiograms, we analyzed ECGs for signs of right ventricular hypertrophy/pressure overload, but found no association with NT-proBNP level. Moreover, the association between NT-proBNP and mortality was present also when adjusting for peripheral edema, a clinical feature of elevated right ventricular filling pressures and for decades known as a strong predictor of mortality in COPD. [34] Several studies have shown that left heart failure is relatively common in COPD patients, [2-4,9,35,36] and we find it reasonable to assume that this contributes to the release of NT-proBNP during AECOPD.

Guidelines provide cut-offs to rule heart failure in or out in stable patients. Attempts have been made to make similar decision limits in the acute setting, [30,37,38] but the grey zone is wide, and such recommendations have not been incorporated in the guidelines. Therefore, in the absence of established cut-offs in the acute setting, we chose to explore the data by analyzing NT-proBNP both as a continuous and categorical variable, categorized by tertiles in the main analysis. Categorization by NT-proBNP concentrations of 1000 and 2500 pg/mL, as suggested by Abroug, [30] also discriminated well. However, we found that a lower cut-off also provided prognostic information, with the lower tertile in our material not being far from the limit to rule heart failure very improbable in the PRIDE algorithm (264.4 and 300 pg/mL, respectively) [37]. We found an association between NT-proBNP concentrations and mortality with both categorizations, as well as analyzed as a logarithmically transformed continuous variable. The association between NT-proBNP tertiles and mortality remained practically unchanged when restricting the analysis to patients with no known history of heart failure, and it is not unlikely that unrecognized left heart failure contributes to the mortality in AECOPD. In our study, 24% of NT-proBNP measurements were above 1800 pg/mL, and 18% were above 2500 pg/mL, indicating a high probability of concomitant left heart failure in one of four to one of five AECOPD admissions [30,37]. The corresponding proportions were 18% and 12% after excluding patients with a history of heart failure.

We found cTnT to be more strongly associated with mortality than NT-proBNP. The pathobiology underlying troponin release is multifaceted. Recently, six potential mechanisms of troponin release have been proposed [39], cardiomyocyte necrosis being the most common. Three alternative mechanisms are cellular release of proteolytic troponin degradation products, increased cellular wall permeability with the release of intact cardiac troponin, and active secretion of vesicles containing cardiac troponin. These may occur during AECOPD, with increased wall stress and hypoxemia, factors that also stimulate the production and release of NT-proBNP. If that were the case, we would expect some degree of correlation between hs-cTnT and NT-proBNP. In previous studies, correlations between NT-proBNP and cTnT, and between BNP and cTnI have been found to be r=0.46 (p<0.001) and r=0.006 (p=0.438), respectively, during AECOPD [15,25]. Different study design as well as different proteins being measured may explain these diverging results. In the present study, the correlation coefficient between hs-cTnT and NT-proBNP (both log-transformed) was 0.34, and the association between hs-cTnT and mortality, as previously published, [14] did not change notably when we took NT-proBNP into account, suggesting a relative pathobiological independence between these biomarkers.

When excluding patients with ischemic ST-T changes or chest pain, the association between NT-proBNP and mortality was moderately attenuated and no longer significant (Table 3, two rightmost columns). This may be partly due to reduced statistical power. However, the point estimate for the strength of the association also was reduced, which suggests that the association between NT-proBNP and mortality was stronger among patients with signs or symptoms of myocardial ischemia. Because of the limited power and the retrospective nature of these analyses, these observations should be interpreted with caution.

Our findings that NT-proBNP and cTnT are independent predictors of mortality, are in accordance with recent observations in both patients with chronic heart failure and stable coronary artery disease, [40,41] as well as in the general population, [42,43] demonstrating that NT-proBNP and cTnT measured with a sensitive assay provide complementary prognostic information. This has not previously been shown in an AECOPD cohort: Medina et al. found 3.9 times increased 1-year mortality in patients with NT-proBNP>459.9 pg/mL, but did not analyze troponin [17]. Chang et al. investigated cTnT (using a conventional assay) and NT-proBNP with regard to mortality after AECOPD, and found that either biomarker above the upper limit of normal was associated with increased 30 days mortality, but not with 12 month mortality [15]. The 1-year mortality in Chang’s study was 18% compared to 30% in the present study. We also report a higher mortality rate than Stolz et al., who observed a 22% mortality rate after 2 years of follow-up and no significant association between BNP and long-term mortality [18]. That we found higher overall mortality may in part explain why we found significant differences also in long-term survival. Moreover, we have included data from repeat admissions in our analysis. It is possible that we have registered some important events during follow-up that were missed by the previous studies. The reasons for the lower survival in our study than in those of Stolz and Chang are uncertain. The age, gender composition, lung function, comorbidities and status on admission appear to be comparable across the studies, and do not explain the survival differences. Our unselected cohort may be more representative of “real life” patients as we did not exclude patients with AECOPD and radiological infiltrates (11%), and this is likely to explain part of the differences in survival. The mortality in our study is within the range that is reported in a 2009 review on COPD exacerbation [44]. In contrast to the report by Chang et al., we found that cTnT, even in modest concentrations, is more strongly associated with mortality than is NT-proBNP.

The survival analysis was done by using an extended Cox regression model with time-dependent covariables. The model is not widely used, but is well described in standard textbooks on survival analysis [29,45]. Importantly, compared to a standard Cox regression model, both the number of observations, the number of events and the observation time is unchanged, so the data are not inflated. Time is counted from inclusion until death or censoring while variables that may vary between admissions are updated at each admission. In our opinion, such models generally reflect the true associations between the recorded variables and the outcome more accurately than standard models. And although these repeat recordings are incomplete (120 of 191 readmissions), we have previously demonstrated that this model has better fit than a model with baseline observations only [14].

The term “frequent exacerbation phenotype” has been introduced over the past years, and previous exacerbation is the best predictor of future exacerbations [27]. As we included patients on admission for exacerbation, it is likely that frequent exacerbators are overrepresented in our material compared to a general COPD cohort. We registered the number of exacerbations in the year prior to inclusion to investigate if frequent exacerbations were associated with NT-proBNP, but failed to find such association (Table 1). Thus we do not believe that exacerbation frequency bias the results of our analyses.

Among the weaknesses of this study is the relatively low number of patients. Nevertheless, we have found a strong and statistically significant association with a clinically important endpoint. Thus, the sample size and thereby the power of the study should be appropriate. Moreover, when comparing our baseline data with those from other studies in the field, [15,46,47] our cohort appears to be representative. Heart failure and pulmonary embolism were not systematically investigated. This would be of interest as it might contribute to troponin release, NT-proBNP increase, and mortality. Echocardiography would provide valuable data, but was beyond the scope of the present study. We did not investigate cause of death. Only four patients were autopsied, and we believe that the cause of death as recorded on the death certificates differentiates poorly between the relevant diagnoses.

## Conclusions

Increasing levels of NT-proBNP are independently associated with long-term mortality after AECOPD, also after multivariable adjustment including the level of cTnT, cephalization on chest radiograph, peripheral edema and gender. Unrecognized concomitant left heart failure may contribute to the increased mortality.

## Competing interests

Prof. Omland has received speakers’ honoraria from Roche Diagnostics, Siemens Healthcare Diagnostics, and Abbott laboratories, manufacturers of assays for B-type natriuretic peptides.

Akershus University Hospital has received research support from Roche Diagnostics and Abbott laboratories, manufacturers of assays for B-type natriuretic peptides.

Doctors Høiseth, Hagve, Brekke and Søyseth have no disclosures.

## Authors' contributions

All authors had full access to the original data and vouch for the completeness and veracity of the data and data analyses. All authors contributed to data interpretation and to the writing of the report, made final decisions on all parts of the report, and approved the final version of the submitted report. PHB, TO and VS designed the study. PHB interviewed and enrolled the patients. TAH measured NT-proBNP and hs-cTnT. AHD and VS confirmed the diagnoses, undertook the statistical analyses and generated tables and figures. VS reviewed the radiographs. AHD and PHB analyzed the ECGs.

## Funding

The study was financed by The Norwegian Association of Heart and Lung Patients through funds from the Norwegian ExtraFundation for Health and Rehabilitation. They had no role in the study design, collection of data, writing of the manuscript or decision to submit for publication.

## Supplementary Material

Additional file 1 **is a word document containing additional information on the patient recruitment and data collection.** There are also some detailed results regarding diagnoses made on later admissions and their relation to the outcome. Click here for file
