# Supplementary material for: NT-proBNP independently predicts long term mortality after acute exacerbation of COPD – a prospective cohort study
Source: Respir Res. 2012 Oct 29;13(1):97. doi: 10.1186/1465-9921-13-97 (PMC3495751; doi:10.1186/1465-9921-13-97)
Supplement: Additional file 1 — is a word document containing additional information on the patient recruitment and data collection. There are also some detailed results regarding diagnoses made on later admissions and their relation to the outcome. [file 1465-9921-13-97-S1.doc]

Høiseth et al.: NT-proBNP Independently Predicts Long Term Mortality after Acute Exacerbation of COPD – a Prospective Cohort Study.

**Additional material - details regarding patient recruitement.**

The study was conducted at Akershus University Hospital (AUH), Norway. AUH is a secondary teaching hospital that, with the exception of severe trauma and patients with ST-elevation myocardial infarctions, receives all medical emergencies from its catchment area. At the time the study was conducted, AUH served a population of about 300.000 inhabitants living in urban, suburban and rural communities.

Patients were included from January 3^rd^ 2005 through to November 30^th^ 2006 and followed until December 31^st^ 2008 or death. All patients admitted with assumed AECOPD were eligible for preliminary inclusion in the emergency room, prior to the emergency physicians’ knowledge of any blood tests. The research fellow contacted the patient on the ward within a day to retrieve written informed consent and medical history. Exclusion criteria were: Age <50 years, metastatic cancer and ECOG performance status grade ≥2, neuromuscular disease with respiratory failure, and non-cooperability. The diagnosis of AECOPD, as defined by the British Thoracic Society in 2004, was later verified by two study doctors by independent review of the hospital records, blinded for the result of the troponin analysis. In case of disagreement, the diagnosis was settled by consensus. Mortality data were gathered from the National Population Registry.

On admission, data were obtained from 234 patients admitted with assumed AECOPD. Out of these, 114 were not included either because the research fellow was absent or because he had not been informed of the patients’ arrival. Of the remaining 120 patients, nine failed to fulfill study entry criteria, leaving 111 consenting patients. Nine patients were excluded as review of their spirometry showed that they did not have COPD. Three patients with COPD were excluded as the primary cause of hospitalisation was pulmonary embolism or pneumothorax, leaving 99 patients for analysis. During the inclusion period, 47 previously included patients were readmitted with verified AECOPD, a total of 191 readmissions. In 120 of these, clinical data were recorded and blood was drawn on admission, thus data from 219 admissions were available for analysis. There was insufficient blood to analyse NT-proBNP in two of these, so 217 observations constitute the study sample.

Spirometries from stable phase only were recorded, and recordings from the outpatient clinic prior to inclusion were preferred. To make the data as complete as possible, we retrieved spirometry reports from collaborating hospitals in some cases. Median time from spirometry (n=88) to inclusion was 179 days (interquartile range 11–416 days), including both before (maximum 1250 days) and after (maximum 341 days) inclusion. In the lung outpatient clinic, spirometry with reversibility testing is the routine, and post bronchodilatation results were used in the analyses. There was no significant difference in NT-proBNP concentrations between the patients who had spirometry available and those who had not. Of the 11 patients who did not have spirometry available, eight died during follow-up.

During the index admissions, no patients were diagnosed with heart failure. Two patients received diagnosis of heart failure during subsequent admissions (hs-cTnT 18 and 71, and NT-proBNP 1740 and 7570). Both patients died. One patient received a discharge diagnosis of an acute coronary syndrome (ACS) during the index hospitalization (hs-cTnT 133, NT-proBNP 104). Four patients received the diagnosis of ACS during subsequent admissions. All five patients survived.

As presented in the main manuscript, the majority of the patients had normal Creatinine concentrations. Using the MDRD formula, 15.5% of the samples had estimated GFR <60 mL/min/1.73 m^2^, and 2% were <30 mL/min/1.73 m^2^. When stratifying for renal function, the mortality rate ratios were 2.4 and 2.2 for creatinine <100 and ≥100 µmol/L, respectively (p=0.882 for interaction).
